# Early self-care rehabilitation of head and neck cancer patients

**DOI:** 10.3109/00016489.2010.532157

**Published:** 2011-05-01

**Authors:** Alexander Ahlberg, Therese Engström, Polymnia Nikolaidis, Karin Gunnarsson, Hemming Johansson, Lena Sharp, Göran Laurell

**Affiliations:** 1 Department of Otolaryngology, Karolinska University Hospital, Stockholm, Sweden; 2 Department of Clinical Sciences, Intervention and Technology (CLINTEC), Karolinska Institutet, Stockholm, Sweden; 3 Department of Speech Pathology, Karolinska University Hospital, Stockholm, Sweden; 4 Department of Clinical Sciences, Intervention and Technology (CLINTEC), Division of Speech and Language Pathology, Karolinska Institutet, Stockholm, Sweden; 5 Department of Physiotherapy, Karolinska University Hospital, Stockholm, Sweden; 6 Department of Oncology, Karolinska University Hospital, Stockholm, Sweden; 7 Department of Oncology-Statistics, Karolinska University Hospital, Stockholm, Sweden; 8 Department of Learning, Informatics, Management and Ethics, Medical Management Center (LIME), Karolinska Institutet, Stockholm, Sweden; 9 Department of Clinical Science, Umeâ University, Umeâ, Sweden

**Keywords:** Quality of life, functional impairment, loss of function, dysphagia, head neck, trismus, neck stiffness, sick leave, speech problem

## Abstract

*Conclusions:* No positive effects of early preventive rehabilitation could be identified. The results do not contradict the proposition that rehabilitation based on self-care can be effective but it is important to establish evidence-based training programs and identify proper instruments for selection of patients and evaluation of intervention. *Objectives:* Patients with head and neck cancer suffer from functional impairments due to intense treatment. In this study, we investigated the effectiveness of an experimental early preventive rehabilitation using hard, objective end points in a nonselective, longitudinal, prospective cohort study. *Methods:* In all, 190 patients were included in the program and received instructions for training before the start of treatment with the aim of reducing swallowing problems and reducing mouth opening and stiffness in the neck. A control group of 184 patients was recruited. *Results:* There was no difference in weight loss and 2-year survival between the two groups. No positive effects concerning functional impairments were found in patient-reported outcome measures.

## Introduction

More attention is being paid to functional impairment and health-related quality of life (HRQOL) in patients with tumor diseases [1]. This has arisen partly from the fact that more patients survive and have a longer expected survival after treatment. The clinical problem of impairment of physical functions in head and neck cancer patients generally appears to be increasing in patients receiving combined modality treatment, probably due to progress in oncologic treatment leading to better survival [2]. Some studies have indicated increased loss of function and more acute side effects if chemotherapy is added to the treatment [3]. One feasible approach that requires evaluation for improvement of HRQOL and reduction of the consequences of functional impairment is rehabilitation.

Swallowing problems are considered to be the most prominent symptom after treatment, and lead to weight loss and probably also reduced HRQOL [4-6]. Another important loss of function is stiffness and pain in the neck and shoulders. These can be related to both radiotherapy and surgery, especially neck dissection, even if the accessory nerve is preserved [7,8].

Traditionally, rehabilitation is initiated when loss of function is already established. An alternative approach might be an early rehabilitation process starting as soon as possible after diagnosis to prevent development of functional impairments or to reduce the extent of these problems [9]. To make this possible, a well-established clinical pathway including the enrolment of patients into a preventive rehabilitation program is crucial, without any delay in the start of treatment [10].

The term cancer rehabilitation has been used in the literature in a wide variety of ways. The lack of a defined role for rehabilitation in head and neck cancer patients encouraged us to develop a self-training program to reduce functional impairment. An experimental clinical development program for early preventive rehabilitation was implemented in 2004 at one of our two radiotherapy sites, as an integrated part of the treatment.

We performed a prospective study on the integration and effectiveness of the early preventive rehabilitation program based on self-care. Our hypothesis was that early preventive rehabilitation of head and neck cancer patients can reduce functional impairment and improve HRQOL, and thereby also affect the survival.

## Material and methods

This study was based on a clinical development program financed by the Swedish Cancer Society and structured as a prospective nonrandomized study comparing two parallel groups, one group undergoing experimental early preventive rehabilitation and the other group not being offered a systematic rehabilitation program. Patients were consecutively included into both groups over 3.5 years and the first year of inclusion, 2004, functioned as a pilot study. Thereby no selection of patients was made.

In Stockholm, all patients with head and neck cancer are treated at one center, which is located at Karolinska University Hospital. Patients are diagnosed at the Department of Otolaryngology and Head and Neck Surgery, where the surgical treatment is also performed. The Department of Oncology has two units for radiotherapy (RT units) at two different locations in Stockholm, the southern RT unit and the northern RT unit.

Patients diagnosed with head and neck cancer in Stockholm from January 1, 2004 to July 31, 2007 who were to receive external beam radiotherapy with curative intent were included in the study. Patients treated at the southern RT unit were offered experimental early preventive rehabilitation and formed the study group, while patients treated at the northern RT unit did not undergo any early rehabilitation and they made up the control group. Patients included in the study group met with a speech pathologist and a physiotherapist who were also part of the research team. Patients included were sent a letter about the study directly after diagnosis, to make sure they received information about the study before the start of treatment.

### Early rehabilitation and evaluation by the speech pathologist

All patients in the study group were examined by the speech/language pathologist (SLP) before radiotherapy and 3 months after completion of therapy. The patients were instructed, both verbally and with written information, on how to perform mobility exercises for the tongue and larynx (Mendelson's maneuver) at least once and preferably twice a day at home during the course of radiotherapy, and for 3 months after termination of treatment. The tongue mobility exercises consisted of five repetitions of extending the tongue as far as possible straight out, up, down, and laterally and then moving the tongue over the whole inside of the oral cavity and teeth. Mendelson's maneuver, holding the larynx at its most superior position for 2-3 s during swallowing, was to be repeated 10 times. At each visit, the patient's swallowing, oral motor function, speech, and voice were examined using clinical screening procedures. At the clinical screening of swallowing, the patient was asked to complete one swallow of two bolus sizes (5 and 15 ml) of four consistencies: thin liquid, thick liquid, paste, and cookie. Movement of the floor of the mouth, hyoid, and thyroid cartilage was evaluated by manual palpation during the act of swallowing. The following swallowing parameters were clinically evaluated: oral manipulation and transport of bolus, presence of aspiration, laryngeal elevation, need for several swallows, delayed initiation of swallowing, and nasal regurgitation. Aspirations were noted as cough, need to clear the throat, wet voice, or sudden breathing difficulties. Patients with a risk of aspiration or those refusing to participate were not given all consistencies at all evaluation points. Voice and articulation were perceptually assessed by the SLP and the tongue motility was assessed with tongue exercises.

### Physiotherapy intervention and evaluation

The patients had an appointment with the physiotherapist (PT) before the start of radiotherapy and follow-ups were performed at 2, 6, and 12 months after termination of treatment. The patients received written and verbal instructions about exercises and stretching of muscles of the head and neck to maintain mobility in the radiotherapy-exposed areas. The exercises for preventing stiffness of the neck consisted of active rotation of the head in both directions, flexion/ extension of the head in a neutral position, and lateral flexions of the head, 3×10 times in each direction. They also involved stretching of the platysma and muscles of the neck. The patients were told that the program should be performed twice a day and performed before, during, and after radiotherapy until follow-up at 6 months, and later if required. In cases of postoperative radiotherapy after neck dissection, the patients were told to pay extra attention to the exercises since the radiotherapy would probably worsen any already restricted head and neck mobility.

The prevention of trismus consisted of exercises with the ‘Acute Medic Jaw Trainer and Stretcher’ (JTS) (www.acutemedic.com, Sweden). The program of active mouth opening was done as active maximal mouth opening assisted with the JTS for 10 × 20 s, twice a day. At follow-ups, the directions could sometimes be changed to a ‘hold and release’ technique, depending on the need and/or compliance of the patient.

The head and neck range of movements and mouth opening (inter-incisial distance between upper and lower left front teeth) was measured before the start of treatment and at all follow-ups. The range of mobility (flexion, extension, rotation, and lateral flexion) in the head-neck region was measured with a Myrin goniometer [11] that measured grades of movement.

### Effect of treatment and rehabilitation

The body weight of the patient was measured routinely at diagnosis and at the follow-ups and registered in the electronic patient files of the hospital. All patients were offered dietary counseling by a dietician before or at the start of radiotherapy and they were given advice about high-calorie diets; when needed, they were offered nutritional supplements. Patients with swallowing problems and weight loss of >5% of their initial body weight and patients with expected nutritional problems caused by advanced tumor (stage IV) were generally given enteral nutrition by the use of a nasogastric tube or percutanous endo-scopic gastrostomy.

Since patients in the control group did not meet the SLP or PT for evaluation of functional loss, all patients in both cohorts who were planned for curative radiotherapy at our weekly tumor meeting were sent a set of questionnaires before the start of treatment and 6 months after termination of treatment, i.e. last surgery or last day of radiotherapy or brachytherapy. The questionnaires used were EORTC-QLQ-C30, EORTC-H&N35 (the European Organization for Research and Treatment of Cancer), HADS (Hospital Anxiety and Depression Scale), and a project-specific questionnaire.

The EORTC-QLQ-C30, the H&N35, and the HADS are validated questionnaires for the aims and study population of the present study [12-15]. The project-specific questionnaire focused on self-reported functional losses and also contained questions about rehabilitation and working ability. The first version was constructed by the research team in 2004 after a literature review, and was tested in a pilot study on a group of patients several months after termination of radiotherapy. Thereafter, the questionnaire was adjusted according to information gained from the patients. The project-specific questionnaire is a Likert-type item scale that captures functional impairments (24 items), patients’ satisfaction with rehabilitation (11 items), return-to-work and sick leave (5 items), demographic and social (5 items), and one open question. The EORTC questionnaires were analyzed according to the EORTC guidelines provided by the EORTC network (http://www.eortc.be/). The following cut-off levels were chosen for HADS based on previous studies [15,16]: normal corresponds to 0-7 points, mild depression/ anxiety corresponds to 8-10 points, moderate depression/anxiety corresponds to 11-15 points, and severe depression/anxiety corresponds to 16-21 points.

### Key measure for integration of the clinical development program

The key measure used to evaluate the integration of the intervention was the length of time between the date of diagnosis and the start of radiotherapy.

### Key measures for improvement and study end points

Weight loss and 2-year survival were chosen as the principal measures of effect. Secondary outcome measures were sick leave, self-reported loss of function, HRQOL, and anxiety/depression.

### Sample size

At the planning stage of the study, it was anticipated that in this population the proportion of patients with no swallowing difficulties after treatment with traditional rehabilitation would be about 50%. To be able to detect an absolute 15% improvement with early preventive rehabilitation, with 80% power, and with 5% significance level, it was estimated that 350 patients in total should be recruited to the study. With this sample size, the predicted precision (95% confidence interval width for the difference in proportions between the two groups) would be about ±10%.

**Figure 1:**
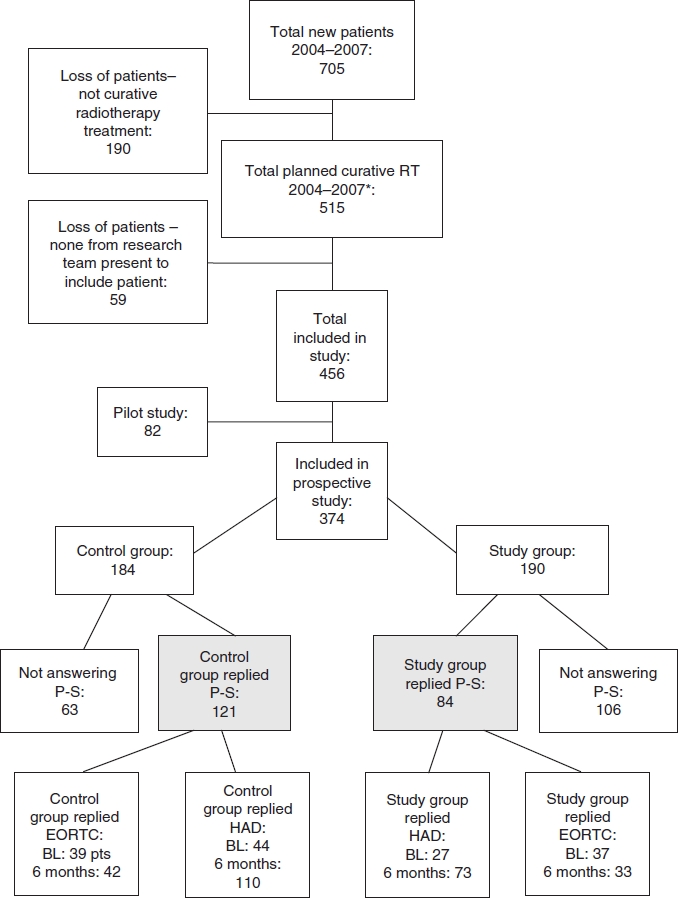
Inclusion of patients and loss of patients. P-S, project-specific questionnaire; BL, baseline (time of diagnosis); EORTC, includes both QLQ-30 and H&N35. 6 months, 6 months after termination of treatment. *Year 2004 includes only patients treated at the southern RT unit and year 2007 runs only until June 30, since the study was terminated after this.

The patient population at the center during the study period and loss of patients are shown in Figure 1. In total, 456 patients were included in the study, 82 in the pilot study and 374 in the prospective study. Of these 374 patients, 205 answered the project-specific questionnaire at 6 months after termination of treatment, 84 in the study group and 121 in the control group. The basic characteristics of the 205 patients were not significantly different from those of the 169 patients not answering the project-specific questionnaire, according to the parameters listed in Table I.

**Table I:** Patient characteristics.

		Patients answering the projectspecific questionnaire (%)	
Characteristics	Total (%)	Study group	Control group
Age (years), mean [SD]	63.7	(12.9)	63.6 (13.1)
Sex			
Male	253 (68)	56 (67)	82 (68)
Female	121 (32)	28 (33)	39 (32)
Stage			
I	40 (11)	9 (11)	17 (14)
II	51 (14)	16 (19)	19 (16)
III	76 (20)	12 (14)	23 (19)
IV	156 (42)	38 (45)	45 (37)
Missing	51 (14)	9 (11)	17 (14)
Site			
Oral cavity	79 (21)	22 (26)	25 (21)
Oropharynx	111 (30)	26 (31)	33 (27)
Epipharynx	10 (3)	2(2)	5 (4)
Hypopharynx	22 (6)	6(7)	4(3)
Larynx	47 (13)	8 (10)	14 (12)
Salivary gland	28 (8)	8 (10)	11 (9)
Nose and sinus	14 (4)	0(0)	6(5)
Other	63 (17)	12 (14)	23 (19)
Radiotherapy			
Dose, mean [SD]	64 (6.8)	65 (5.7)	63 (7.3)
Preoperative RT	116 (31)	26 (31)	36 (30)
Postoperative RT	131 (35)	33 (39)	52 (43)
Only RT	122 (33)	25 (30)	32 (27)
Surgery			
Any surgery	254 (68)	59 (70)	89 (74)
No surgery	120 (32)	25 (30)	32 (26)
Major surgery		6(7)	12 (10)
Chemotherapy	86 (23)	10 (12)	35 (29)
Number of patients	374		84

Patient characteristics for the total patient material included in the prospective study and separately for the study and control groups are listed in Table I. The basic characteristics of the two groups did not show any significant differences and the groups were well matched. There was a significant difference in the number of patients who received chemotherapy between the RT units (*p* = 0.004).

### Diagnosis and treatment

Patients were diagnosed by clinical examination, CT scan of the head and neck and the thorax, ultrasound-guided fine-needle aspiration of metasta-ses in the neck, and an endoscopic examination under general anesthesia.

Radiotherapy was given at the two different radiotherapy sites in Stockholm and dose plans were constructed according to local guidelines. Chemotherapy was given either as induction treatment or as concomitant treatment. Brachytherapy was mainly used for patients with tumors in the base of the tongue and for some cases with tumor in the mobile part of the tongue or the floor of the mouth.

Surgery was performed according to local guidelines at the Department of Otolaryngology and Head and Neck Surgery at Karolinska University Hospital following the same guidelines for both groups.

### Follow-up

All patients were followed up for at least 2 years according to set guidelines, with visits every third month for the first 2 years and thereafter every 6 months.

### Statistical methods

Survival time was calculated from the date of the multiprofessional treatment meeting to the date of death, or until the common date for the end of survival follow-up, August 1, 2009. Survival was estimated according to the Kaplan-Meier technique and differences in survival times were tested using log rank test. Confidence interval for the reported survival differences refers to the fixed 2-year time point.

Continuous variables were analyzed using linear regression models. In these models, intervention is included in the model as a categorical variable taking the value of 0 for the control group and 1 for the study group. For some continuous outcome variables, baseline values were available. In these cases, models were estimated including the baseline values as well as the intervention variable. Differences in categorical variables were tested using Fisher's exact test. Binary outcomes were modeled using ordinary logistic regression models. For ordinal variables, distributional comparisons were performed using the Mann-Whitney U test and the ordinal outcomes were modeled using the proportional odds model. The exponentiated intervention coefficient in these models can be interpreted as the ratio (between the study group and control group) of the odds of being in a higher (worse) rather than lower (better) category. All effects from the regression models are presented together with 95% confidence intervals.

### Ethical considerations

The Research Ethics Board of Stockholm approved the study by judging that no ethical approval was required as it was identified as a clinical development program (entry no.: 2005/767-31/1-4). The study was performed according to the Declaration of Helsinki.

## Results

### Time between diagnosis and start of treatment

All patients undergoing radiotherapy at the site for intervention (the southern RT unit) could be included in the clinical development program. There was no difference in time from diagnosis to start of treatment between the study group (25.9 days, SD = 30.5) and the control group (25.9 days, SD = 30.4) (*p* = 0.99).

### Weight loss and survival

There was no significant difference in weight loss (defined as the change in weight from diagnosis to 6 months after treatment) between the study group (-5.9 kg) and the control group (-6.2 kg), see Table II. The difference in the incidence of patients with weight loss of > 10%, although higher in the control group, was not significant.

**Table II:** Comparison of outcome between study group and control group concerning weight loss and working ability.

	Intervention study (%)				
Characteristics	n*	Study group	Control group	*p* value	Difference (95% confidence interval)
Weight loss*	197				
Stable or increase		17 (21)	15 (13)		
Decrease <;5%		18 (22)	27 (24)		
Decrease 5–10%		27 (33)	30 (26)		
Decrease >10%		21 (25)	42 (37)	0.23	-
Mean weight loss† in kg [SD]		−5.8 (7.8)	−6.2 (5.8)	0.68	0.4 (−1.5 to 2.3)
On sick leave at baseline	22				
Situation unchanged at 6 months		7 (64)	7 (64)		
Situation better at 6 months		4(36)	4(36)	1.0	-
Not on sick leave at baseline	133				
Situation unchanged at 6 months		29 (55)	64 (80)		
Situation worse at 6 months		24 (45)	16 (20)	0.003	25.2 (9.3 to 41.3)
Total number of patients	205	84	121		

Calculations of percentage and SD included patients with zero-values.

* Number of patients included in the analysis.

† The difference in weight loss after controlling for baseline weight was not significant (*p* = 0.42) and was estimated to be 0.7 (−1.0 to 2.4).

Overall 2-year survival for all patients in the study (*n* = 374) was 76% (95% CI: 71-80). There was no significant difference in 2-year survival between the study group (*n* = 190) and the control group (*n* = 184) (log rank, *p* = 0.49).

### Working ability

As shown in Table II, a significantly higher number of patients in the control group who had been working before diagnosis had returned to work by 6 months after treatment, i.e. more patients in the study group were still on sick leave and had not returned to work.

### Swallowing

Table III illustrates that patients in the control group reported significantly less swallowing difficulties than those in the study group, with a proportional odds ratio (OR) of 2.3 (95% CI: 1.3-4.0). In the control group, 58% answered that they could swallow all consistencies of food compared with 35% in the study group (*p* < 0.001; data not shown in tables). There were a significantly higher number of patients using high kilocalorie/protein supplement in the study group (60%) than in the control group (32%) (*p* < 0.001). The total incidence of reported swallowing problems was 61% (114/185).

**Table III:** Comparison of outcome between study group and control group concerning self-reported loss of function 6 months after treatment.

	Intervention study (%)			
Characteristics	*n*	Study group	Control group	*p* value
Do you have PEG?	167			
1. Yes		12 (18)	15 (15)	
2. No		46 (69)	71 (71)	
3. No, but I used to have		9 (13)	14 (14)	0.88
Do you feel you have recovered after your treatment?	196			
1. Yes, completely		4 (5)	18 (16)	
2. Yes, partly		35 (42)	51 (45)	
3. No		41 (49)	36 (32)	
4. Don't know		3 (4)	8 (7)	0.016
Swallowing difficulties*	185			
1. Not at all		20 (26)	51 (47)	
2. A little		27 (36)	35 (32)	
3. Quite a bit		23 (30)	16 (15)	
4. Very much		6 (8)	7 (6)	0.003
Chewing difficulties*	186			
1. Not at all		28 (38)	45 (40)	
2. A little		21 (29)	31 (27)	
3. Quite a bit		18 (25)	22 (20)	
4. Very much		6 (8)	15 (13)	0.94
Reduced ability to open mouth*	187			
1. Not at all		25 (34)	56 (50)	
2. A little		20 (27)	28 (25)	
3. Quite a bit		21 (28)	23 (20)	
4. Very much		8 (11)	6 (5)	0.018
Speech problems*	184			
1. Not at all		20 (28)	54 (48)	
2. A little		26 (36)	39 (35)	
3. Quite a bit		16 (22)	11 (10)	
4. Very much		10 (14)	8 (7)	0.001
Stiffness in the neck/shoulders*	189			
1. Not at all		23 (31)	33 (29)	
2. A little		27 (36)	47 (41)	
3. Quite a bit		20 (27)	22 (19)	
4. Very much		5 (7)	12 (11)	0.98

* Data treated as ordinal.

No difference in the incidence of dryness of the mouth was found. The total incidence of patients reporting any level of dryness of the mouth was 92% (184/201) and the incidence of those reporting that they had ‘very great’ problems with dryness of the mouth was 41% (82/201).

### Speech problems

There was a significant difference in the extent of speech problems, with an OR of 2.5 (95% CI: 1.4-4.4). In the study group, there was an increased risk of being at a worse level (Table III).

### Trismus and stiffness of the neck

In the total material, the incidence of chewing problems was 61% (113/186); 57% (106/186) reported a reduced ability to open the mouth and 70% (133/189) reported stiffness of the neck and shoulders 6 months after termination of treatment. There was an OR of 1.9 (95% CI: 1.1-3.3) with an increased risk of reduced ability to open the mouth in the study group relative to the control group. However, there was no difference between the groups concerning chewing of food. There was also no difference in stiffness of the neck and shoulders (Table III).

### HRQOL and anxiety / depression

These parameters are described in Table IV. Response frequency was lower in EORTC than in the project-specific questionnaire. No parameters in the EORTC or the HADS showed any significant difference between the two groups at 6 months after termination of treatment. At 6 months after treatment, the total incidence of depression at any level (mild to severe) was 27% (50/183).

**Table IV:** Comparison between study group and control group concerning global health (EORTC QLQ-30) and depression and anxiety (HAD-S) measured 6 months after treatment.

	Intervention study (%)			
Characteristics	*n*	Study group Mean (SD)	Control group Mean (SD)	*p* value
HAD-S				
Depression	183			
1. Normal (0–7)		54 (74)	79 (72)	
2. Mild (8–10)		11 (15)	16 (15)	
3. Moderate (11–15)		5 (7)	14 (13)	
4. Severe (16–21)		3 (4)	1 (1)	0.75
Anxiety	189			
1. Normal (0–7)		53 (70)	81 (72)	
2. Mild (8–10)		8 (11)	13 (12)	
3. Moderate (11–15)		11 (15)	17 (15)	
4. Severe (16–21)		4 (5)	2 (2)	0.66
EORTC QLQ-30 Global health status	75	56.6 (23.4)	61.4 (23.0)	0.29

## Discussion

Disability dominated by reduced functional capacity is common in head and neck cancer patients after treatment. We have implemented a clinical development program based on self-care, aimed at rehabilitating patients treated for head and neck cancer with radiotherapy combined or not combined with surgery, chemotherapy, or brachytherapy. The clinical development program concentrates on maintaining swallowing function and mobility of the jaw and neck. Our hypothesis was that an improvement can be achieved by invoking early preventive rehabilitation that starts before treatment and is conducted by a speech/language pathologist and a physiotherapist [10]. The present study is a nonse-lective, longitudinal, prospective cohort study evaluating the effect of the experimental early preventive rehabilitation.

Rehabilitation has been tested clinically and experimentally and is an area of great interest but a gold standard is still not available. There are different ways to perform experimental early rehabilitation. One common approach is to select a smaller group of patients based on their specific needs and to provide them with an intense program with frequent evaluations [17]. An alternative model used in the current study is to include all possible patients without selection before treatment and increase the patient activation and involvement by self-care. Another motive for research on preventive rehabilitation based on self-care is resource utilization aiming to provide a cost-effective alternative that could be used in treatment decisions.

For the majority of patients, delay in treatment would be associated with a less favorable prognosis and it is therefore most important that preventive rehabilitation should not affect the start of treatment [18]. An important finding is that no delay in the start of treatment was found in patients undergoing early rehabilitation, indicating that a rehabilitation program like this could be effectively integrated into the existing clinical routines.

The instruments for evaluation and outcome variables in earlier studies have been variable. Here the critical end points were weight loss and 2-year survival, which are strong objective values that can be continuously followed through the patients medical files. No difference between the study group and the control group was found concerning weight loss or 2-year survival. It might be argued that the applied primary outcome measure weight loss might be too rigid and not fully relevant for determining the effectiveness of early preventive rehabilitation. The local nutritional guidelines might have influenced the primary outcome measure weight loss more than the rehabilitation program focused on self-care regarding swallowing. It is also less probable that survival would be affected by this kind of rehabilitation program. However, survival is a strong objective parameter that is often discussed in association with quality of life and reflects not only cancer death but also non-cancer mortality associated with treatment.

The analysis of the patient-reported outcome measures also showed no positive effects of the rehabilitation program. Thus, an intervention using early preventive rehabilitation based on self-care was not superior to conventional follow-up in consecutive head and neck cancer patients. Furthermore, it was found that patients who received early rehabilitation scored significantly lower on the project-specific self-report questionnaire concerning swallowing and jaw mobility. It should be noted that significantly more patients in the control group received chemotherapy and might therefore be expected to suffer from more morbidity than the study group.

The results of the current study cannot therefore confirm the observations of Carroll et al. [4] in a retrospective case control study of nine patients who underwent pretreatment swallowing exercises. This discrepancy may be accounted for by the fact that they used a limited sample size and selected patients for training of the swallowing function. Nor could the current study confirm the positive outcome of pretreatment swallowing education and exercise found by Kulbersh et al. [9] in a study involving 37 patients using the MD Anderson Dysphagia Inventory (MDADI) questionnaire. A possible interpretation of the findings in the current study is that after detailed information and self-care, patients in the study group were more aware of swallowing problems and the importance of nutritional intake before therapy, and therefore had higher expectations concerning their functional status than the control group.

It is believed that a considerable number of patients with head and neck cancer have a low HRQOL already before the onset of symptoms, and could therefore be a complicated and complex group to rehabilitate [19]. The two groups of patients in the present study were similar in many aspects, although we have not analyzed socioeconomic factors thoroughly. The employment of other social variables might have been important parameters to analyze, since there were more patients who had worked before diagnosis still on sick leave in the study group than in the control group 6 months after treatment.

The clinical development program was running for 3.5 years and was thereafter implemented as clinical routine before the data from the current study had been thoroughly analyzed. As a worse outcome was revealed for the rehabilitation group in some domains in the project-specific questionnaire one may question a continuation of preventive rehabilitation in a clinical setting. However, there are no obvious indications that the experimental early rehabilitation program itself aggravated the functional impairments. It is well known from several earlier rehabilitation studies in different medical fields that rehabilitation outcomes can be hard to assess. Nevertheless rehabilitation based on self-care in the present form does not seem to be a promising strategy to reduce disability in the majority of patients that are treated for head and neck cancer. The idea of totally abandoning the concept of preventive rehabilitation in this patient group is not justified by the results in this study. Instead there is now a need to take a further step and find new approaches for preventive rehabilitation with the goal of maintaining swallowing function, speech, and motility of the jaw and neck.

As far as we are aware, there have been no previous studies evaluating self-care for early preventive rehabilitation in a large sample of unselected head and neck cancer patients. However, as stated in previous reports, this is much needed. In the light of previous studies of swallow exercise programs showing encouraging outcomes there are two major considerations regarding the negative results of the early rehabilitation program. First, the determinants of rehabilitation may have been insufficient to detect minor positive effects of the program. Second, the model for early rehabilitation based on self-care before, during, and after treatment with just a few follow-ups may have produced an ambivalent attitude in patients. It could be speculated that another design for early preventive rehabilitation might have had a favorable outcome. The lack of positive results justifies reconsideration of a randomized controlled trial of early rehabilitation of head and neck cancer patients. We recommend that such a study should use more regular surveillance and physical evaluation of patients in both the study group and the control group.

## Conclusion

An early rehabilitation program including head and neck cancer patients in general was introduced without extending the period between diagnosis and treatment. However, this study could not show any positive effect of early preventive rehabilitation based on self-care. It is important to identify proper instruments for evaluation of early rehabilitation.
